# ErbB4 promotes inhibitory synapse formation by cell adhesion, independent of its kinase activity

**DOI:** 10.1038/s41398-021-01485-6

**Published:** 2021-06-29

**Authors:** Bin Luo, Ziyang Liu, Dong Lin, Wenbing Chen, Dongyan Ren, Zheng Yu, Mingtao Xiong, Changqin Zhao, Erkang Fei, Baoming Li

**Affiliations:** 1 School of Life Sciences, Nanchang University, Nanchang, China; 2 Institute of Life Science, Nanchang University, Nanchang, China; 3 Department of Psychology and Institute of Brain Science, School of Education, Hangzhou Normal University, Hangzhou, China

**Keywords:** Molecular neuroscience, Schizophrenia

## Abstract

The precise control of the nervous system function under the vitality of synapses is extremely critical. Efforts have been taken to explore the underlying cellular and molecular mechanisms for synapse formation. Cell adhesion molecules have been found important for synapse assembly in the brain. Many trans-adhesion complexes have been identified to modulate excitatory synapse formation. However, little is known about the synaptogenic mechanisms for inhibitory synapses. ErbB4 is a receptor tyrosine kinase enriched in interneurons. Here, we showed that overexpressing ErbB4 in HEK293T cells induced gephyrin or GABA_A_R α1 puncta in co-cultured primary hippocampal neurons. This induction of ErbB4 was independent of its kinase activity. K751M, a kinase-dead mutant of ErbB4, can also induce gephyrin or GABA_A_R α1 puncta in the co-culture system. We further constructed K751M knock-in mice and found that the homozygous were viable at birth and fertile without changes in gross brain structure. The number of interneurons and inhibitory synapses onto pyramidal neurons (PyNs) were comparable between K751M and wild-type mice but decreased in ErbB4-Null mice. Moreover, ErbB4 can interact in trans with Slitrk3, a transmembrane postsynaptic protein at inhibitory synapses, through the extracellular RLD domain of ErbB4. The deletion of RLD diminished the induction of gephyrin or GABA_A_R α1 puncta by ErbB4. Finally, disruption of ErbB4–Slitrk3 interaction through neutralization of Slitrk3 by secretable RLD decreased inhibitory synapses onto PyNs and impaired GABAergic transmission. These results identify that ErbB4, as a cell adhesion molecule, promotes inhibitory synapse formation onto PyNs by interacting with Slitrk3 and in a kinase-independent manner, providing an unexpected mechanism of ErbB4 in inhibitory synapse formation.

## Introduction

GABAergic inhibitory interneurons (INs), comprising 15% of neocortical neurons in the central neural system (CNS), play an important role in maintaining the excitatory and inhibitory balance (E/I balance) [1–3]. An appropriate E/I balance is critical to brain functions, and its imbalance is believed to be a fundamental contribution to neuropsychiatric and neurological disorders, such as schizophrenia, autism, and epilepsy [4–6]. As the dominant source of inhibition throughout the CNS, GABAergic synapses provide a vital inhibitory drive to control neuronal circuitry excitability. INs form inhibitory synapses onto excitatory pyramidal neurons (PyNs) and control their activity by releasing GABA. Synapse formation requires transsynaptic interaction between specific cell adhesion proteins. For example, the formation of excitatory synapses involves β-neurexin (NRXNβ) and neuroligin1 (NLGN1), EphB2 and EphrinB3, LAR and NGL-3 [7–11]. However, most studies focus on excitatory synapse formation, but not GABAergic synapse formation. Thus, it is critical to understand the molecular determinants of GABAergic synapse formation. Recent data suggest a role of two postsynaptic proteins NLGN2 and Slitrk3 in inhibitory synapse assembly [11–15].

ErbB4, a receptor tyrosine kinase, is expressed specifically in INs that can be activated by neuregulins (NRGs) [16–19]. The NRG1-ErbB4 signaling has a vital role in GABAergic circuit development, including INs migration and differentiation as well as GABAergic transmission [16, 18–24]. At the subcellular level, ErbB4 locates at the postsynaptic compartments of excitatory synapses onto INs [16, 23] and has thus been implicated in the formation and maturation of these synapses [22, 25–27]. Although ErbB4 is also present in axon terminals of INs, its role in the formation of inhibitory synapses is not well understood. Recently, Del Pino I et al. [26] found that ErbB4 loss of function in vivo reduced the number of inhibitory synapses onto cortical PyNs. However, the underlying mechanisms of this effect remain unclear.

Here we investigated how ErbB4 regulates the formation of inhibitory synapses onto PyNs. Expressing ErbB4 in HEK293T cells was able to induce gephyrin or α1 subunit of GABA_A_ receptor (GABA_A_R α1) puncta in the dendrites of cultured neurons, indicative of a role of ErbB4 in the postsynaptic differentiation of inhibitory synapses onto PyNs. Interestingly, this effect was independent of its kinase activity in vitro. To test this hypothesis in vivo, we generated a knock-in mutant strain line, K751M, which expresses kinase-dead ErbB4. Surprisingly, we found the number of inhibitory synapses and INs were similar between K751M and control mice, suggesting that the kinase activity of ErbB4 is not necessary for these two events. Furthermore, we identified the Slitrk3 as the functional postsynaptic receptor for ErbB4. Extensive induction, disruption experiments in vitro and in vivo support the idea that ErbB4, as a cell adhesion molecule, controls functional inhibitory synapse formation via trans interaction with postsynaptic Slitrk3.

## Materials and methods

### Animals

Heart-rescued ErbB4 knockout mice (termed as Null mice in this study), *GAD67*::GFP mice were described previously [28–31]. ErbB4-K751M mice were generated by CRISPR genome editing technology (Bioray Laboratories Inc., Shanghai, China). To generate the targeting construct, a specific gRNA was designed to recognize the mutant site area: 5′-AAACGTGGCTATAAAGATCCTC-3′. And single-stranded oligo-deoxyribonucleotides (ssODN) was designed for the homology-directed repair (HDR): 5′TATTTGGGTGCCTGAAGGTGAAACAGTGAAAATCCCTGTCGCAATTATGATCCTCAATGAAACAACTGGCCCCAAAGCCAACGTGGAGTTCATGGATGT-3′. The CRISPR components (cas9/sgRNA, ribonucleoproteins complex, and ssODN) were microinjected into one-day-old mouse zygotes and transferred to pseudopregnant foster mothers. 7 weeks later, biopsy samples from the pups derived from the microinjected zygotes, were collected and genotyped with primers (Fw: 5′GCCATGAACTTGAAGGAGAGTG-3′; Rv: 5′GGACCTACTGTCATCACCATCA-3′). PCR results were verified by sequencing. ErbB4-K751M; *GAD67*::GFP and ErbB4-Null; *GAD67*::GFP mice were generated by crossing K751M or Null with *GAD67*::GFP mice. All mice we used were under C57BL/6 background. All experiments were performed with male mice. Mice were housed in a room at 22 °C in a 12 h light/dark cycle with free access to food and water ad libitum. Experimental procedures were approved by the Institutional Animal Care and Use Committees of Nanchang University.

### Reagents, antibodies, and plasmids

Chemicals were purchased from Sigma-Aldrich unless otherwise indicated. Primary antibodies information as follow: mouse anti-MAP2 (1:500 for staining; Millipore; MAB3418), rabbit anti-gephyrin (1:200 for staining, 1:500 for blotting; SYSY; 147018), mouse anti-VGAT (1:500 for blotting; SYSY; 131011), mouse anti-synaptophysin (1:500 for blotting; Dako; M7315), rabbit anti-GAD67 (1:500 for blotting; Thermos Fisher; PA5-21397), rabbit anti-actin (1:1000 for blotting; Santa Cruz; sc-1616-R), Flag-beads (for co-IP; Sigma; A2220), mouse anti-myc (1:1000 for blotting; DSHB; 9E10), rabbit anti-flag (1:2000 for blotting; Sigma; F7425), mouse anti-ankyrin-G (1:500 for staining; NeuroMab; N106/36), rabbit anti- GABA_A_R α1 (1:500 for staining; SYSY; 224203), rabbit polyconal anti-PV (1:1000 for staining; Swant; PV 25), mouse monoclonal anti-NeuN (1:500 for staining; Neuromis; M022122), mouse anti-GFP (1:1000 for staining and blotting; Invitrogen; A-11120), rabbit anti-ErbB4 (1:1000 for blotting; Cell Signaling Technology; 4795), rabbit anti-phoshpo-Y1284-ErbB4 (p-ErbB4, 1:1000 for blotting; Cell Signaling Technology; 4757 S).

To generate pFlag-ECD-IgG, pFlag-RLD1-IgG, pFlag-FLD-IgG, pFlag-RLD2-IgG, and pFlag-GFRD-IgG plasmids, the different domain fragments were amplified by PCR with pFlag-ErbB4-WT as template and inserted into pFlag-IgG backbone via NotI and XbaI sites. ECD, RLD1, FLD, RLD2, and GFRD were amplified with following primers: ECD: 5′ATAAGAATGCGGCCGCACAGTCAGTGTGT-3′ and 5′TGCTCTAGAGCATGGCCCGTCCA-3′; RLD1: 5′ATAAGAATGCGGCCGCAAACTGTGAGGTT-3′ and 5′TGCTCTAGAGCAATATCTTGCCA-3′; FLD: 5′ATAAGAATGCGGCCGCATGTGGACGTTGC-3′ and 5′TGCTCTAGAGCTTTTGGGCAAAT-3′; RLD2: 5′ATAAGAATGCGGCCGCAAACTGTACCAAG-3′ and 5′TGCTCTAGAGCGAAGAGTGTTGT-3′; GFRD: 5′ATAAGAATGCGGCCGCAGTGTGCAACCAT-3′ and 5′TGCTCTAGAGCAATGCAGTCATG-3′. K751M or ΔRLD mutation was generated by QuikChange site-directed mutagenesis (Agilent) with primers: for K751M, 5′CCAGTTGTCTCATTAAGAATCATAATAGCCACAGGAATCTTCA-3′ and 5′TGAAGATTCCTGTGGCTATTATGATTCTTAATGAGACAACTGG-3′; for ΔRLD, 5′CATGGGTTCCGATTTCATAGTACTTGCGCAAGGCT-3′ and 5′AGCCTTGCGCAAGTACTATGAAATCGGAACCCATG-3′ to delete RLD1; 5′CTGGTTGATTGTGCTTATGAATTTGTCAATGTTACTGGAATCCACA-3′ and 5′-TGTGGATTCCAGTAACATTGACAAATTCATAAGCACAATCAACCAGA-3′ to delete RLD2. For pUltra-EGFP-P2A-flag-RLD-IgG plasmid, RLD-IgG was inserted into pUltra (Addgene, #24129) backbone via XbaI and BamHI sites. The authenticity of all constructs was verified by DNA sequencing and western blotting analysis.

### PSD fractionation

The postsynaptic density (PSD) fractionation was performed as described previously [32]. In brief, six male adult mice per group were used and mouse brains were homogenized in 4-(2-hydroxyethyl)-1-piperazineethanesulfonic acid (HEPES) buffer (0.32 M sucrose, 4 mM HEPES [pH 7.4]). The homogenate (Hom.) was centrifuged to remove the pelleted nuclear fraction (P1), and the supernatant was centrifuged again to yield a crude synaptosomal fraction (P2). The washed P2 fraction (P2’) was subjected to hypoosmotic shock and lysis before centrifugation again. After centrifugation, the supernatant (S3) was centrifuged to yield the pellet enriched with synaptic vesicle protein (SV fraction); and the resultant pellet (P3) was resuspended and centrifuged in a sucrose gradient to yield the synaptic plasma member (SPM) fraction. The SPM fraction was incubated with 1% Triton X-100 in 50 mM HEPES (pH 8.0) at 4 °C for 30 min and subjected to centrifugation to yield the supernatant (presynaptic membrane fraction, Pre) and the pellet (PSD).

### Cell culture, transfection, and protein purification

Human embryonic kidney (HEK) 293 T cells were cultured in Dulbecco’s modified Eagle’s medium (DMEM) (Gibco) supplemented with 10% fetal bovine serum (FBS) (Gibco). Transient transfection was performed using polyethylenimine (PEI) (Sigma; 408727), as described before [33]. In brief, cells were cultured in 100 mm dishes and at ∼70% confluence were incubated with precipitates formed by 5 μg of plasmid DNA and 280 μL of 0.05% (wt/vol) PEI. Cells were harvested 24~48 h post transfection.

For protein purification, the medium of transfected cells was changed into pure DMEM on the second day. Cells were continuously cultured for 2~3 days. All medium was collected and added with protein A/G beads for immunoprecipitation (IP). Beads were washed with tris-buffered saline (TBS) (50 mM Tris HCl, 150 mM NaCl, pH 7.4) buffer three times, then five packed beads volumes of 0.1 M glycine HCl buffer (pH 3.0) were added. Samples were incubated with gentle shaking for 5 min at room temperature. Centrifuge at 2500 rpm for 5 min to harvest supernatant and add 10 µl of 0.5 M Tris HCl, pH 7.4, with 1.5 M NaCl. After quantification of protein concentration, the products were stored at −80°C.

Cultures of primary hippocampal neurons were prepared from embryonic day (E) 18.5 Sprague–Dawley rats or mice as described previously [32]. In brief, hippocampi were isolated and kept separate from one another in Hank’s balanced salt solution on ice. Following digestion in 0.25% trypsin plus 0.1 mg/mL DNase I (one hippocampus in 1 mL) at 37°C for 20 min. Dissociated cells were resuspended in plating media (DMEM supplemented with 10% FBS) and plated at a density of 1 × 10^5^ or 2 × 10^5^ per well onto poly-D-lysine-coated 20 mm coverslips (WHB) in 12-well plates (Corning). Cells were incubated for 4 h before replacing with maintenance medium [neurobasal medium (Gibco) supplemented with 2% B-27 supplement (Gibco), 1% GlutaMax (Gibco), and 1% penicillin/streptomycin (Gibco)]. Neurons were maintained at 37°C in 5% CO_2_, with half of the medium changed every 2~3 days.

### Artificial synapse formation assays

Experiments were performed as previously described [34, 35]. In brief, primary cultured hippocampal neurons were seeded on coverslip at a density of 5 × 10^4^/mL. Eight days later, HEK293T cells were co-transfected with EGFP and ErbB4-WT, ErbB4-K751M, Neurexin1α (NRXN1α), or control empty vector by PEI. After 48 h, transfected HEK293T cells were trypsinized and resuspended with a maintenance medium. Transfected HEK293T cells were seeded at a density of 3 × 10^4^ per coverslip onto cultured hippocampal neurons at 10 days in vitro (DIV10) for 2 days. Then all cells were fixed and co-stained with gephyrin or GABA_A_R α1 and MAP2 antibodies. All images were acquired with a confocal microscope (Olympus FV1000). The contours of transfected HEK293T cells were chosen as the region of interest to quantify. The fluorescence intensity of gephyrin or GABA_A_R α1 puncta was normalized to HEK293T cell area by using ImageJ (NIH).

### IP and western blotting

For co-immunoprecipitation (co-IP), transfected HEK293T cells were lysed in IP buffer containing: 20 mM Tris, pH 7.6, 50 mM NaCl, 1 mM EDTA, 1 mM NaF, 0.5% Nonidet P-40 (vol/vol), with protease and phosphatase inhibitors. Samples were centrifuged at 12,000 × *g* for 20 min at 4°C to remove debris. Lysates (1~2 mg) were incubated with the corresponding antibody (1~2 μg) at 4°C for either 3~4 h or overnight and then incubated with 10~15 μL Protein A/G magnetic agarose beads (Pierce) at 4°C for 1 h. Samples were washed with IP buffer and resuspended in sodium dodecyl sulphate (SDS) sample buffer. Then the samples were subjected to western blotting.

For protein expression detection, tissues were homogenized in phosphate-buffered saline (PBS) plus protease and phosphatase inhibitors. Then the homogenates were lysed in an equal volume of 2 × IP assay buffer [0.2% SDS (wt/vol), 1% sodium deoxycholate (wt/vol) and 2% Nonidet P-40 (vol/vol) in PBS] plus protease and phosphatase inhibitors. Lysates were centrifuged at 12,000 × *g* for 20 min at 4°C to remove debris. The supernatants were subjected to Bradford assay (Pierce) to measure protein concentration and diluted in SDS sample buffer.

Protein samples (10~20 μg) were resolved by SDS–polyacrylamide gel electrophoresis and transferred to the polyvinylidene difluoride membrane (Millipore). The membrane was immunoblotted with primary and secondary antibodies, and immunoreactive bands were visualized by enhanced chemiluminescence under the gel documentation system (Bio-Rad). Densitometric quantification of protein band intensity was performed by using ImageJ.

### Nissl staining

Brain sections (40 μm) were harvested from adult mice. Brain sections were rehydrated through 100, 90, 75, and 50% alcohol to distilled water. Afterward, the sections were stained in cresyl violet solution for 5 min and then dehydrated through distilled water to 50, 75, 90, and 100% alcohol, cleared in xylene two times. Finally, the slides were mounted with a resinous medium and analyzed under a bright-field microscope (Olympus FSX100).

### Immunohistochemistry

Mice were acutely anesthetized and transcardially perfused with 1× PBS followed pre-cold 4% paraformaldehyde (PFA). Brains were harvested and post-fixed in 4% PFA at 4°C overnight. Brains were embedded with 2% agarose gel and sectioned into serial 40-μm thick coronal slices by vibratome (Leica VT1000S). Sections were rinsed in 0.5 M TBS for 10 min, then blocked with 10% donkey serum and 0.5% Triton X-100 (diluted in 0.5 M TBS) for 1 h at room temperature. After that, slices were incubated with primary antibodies overnight at 4°C. After washing with TBS three times, slices were incubated with secondary antibodies for 2 h at room temperature and washed with TBS three times again. Finally, slices were mounted with AQUA-MOUNT (Lerner laboratories; 13800).

For the immunostaining of primary cultured neurons, neurons were fixed with 4% PFA for 15 min. Then, neurons were washed three times with PBS. Next, neurons were incubated with primary antibodies overnight at 4°C. After washing three times with washing buffer (contains 20 mM phosphate buffer and 0.5 M NaCl), Neurons were incubated with secondary antibodies for 1 h at room temperature. Neurons were washed three times in PBS before being mounted on Superfrost Plus Microscope Slides (Fisher Scientific cat# 1255015). All images were acquired with confocal microscopy (Olympus FV1000).

To quantify PV+ boutons onto somas or AISs, Z-plane images of individual soma or AIS were acquired with a ×60/1.49 NA oil-immersion objective (Olympus), 0.2 μm/step. All quantifications were analyzed with *Z* axis projection images. Besides, all quantifications were analyzed by investigators blind to genotypes or cell conditions.

### Cell aggregation assay

Experiments were performed as previously described [8]. HEK293T cells were individually transfected with the expression vectors as indicated in the figures. After 48 h, the cells were detached using 1 mM ethylenediaminetetraacetic acid (EDTA) in PBS, mixed, and incubated under gentle agitation at room temperature in DMEM containing 10% FBS, 50 mM HEPES-NaOH, pH 7.4, 10 mM MgCl_2_, and 10 mM CaCl_2_. The extent of cell aggregation was assessed at 90 min by removing aliquots, spotting them onto culture slides (BD Falcon), imaging by epifluorescence microscopy. The resulting images were analyzed by counting the number and size of arbitrary values for the field using ImageJ.

### Electrophysiological analysis

Experiments were performed as previously described [36]. Male mice (P30) were anesthetized with isoflurane and killed by decapitation. Brains were quickly removed to ice-cold oxygenated (95% O_2_/5% CO_2_) cutting solution containing: 120 mM choline chloride, 2.5 mM KCl, 7 mM MgCl_2_, 0.5 mM CaCl_2_, 1.25 mM NaH_2_PO_4_, 26 mM NaHCO_3_, and 25 mM glucose. Lamellar 300 μm slices of the hippocampus using VT1000S Vibratome (Leica Microsystems). The slices were recovered in oxygenated artificial cerebrospinal fluid (ACSF) for 30 min at 32°C and maintained at room temperature (25 ± 1°C) for an additional 1 h before recording. The ACSF containing: 124 mM NaCl, 2.5 mM KCl, 2 mM MgSO_4_, 2.5 mM CaCl_2_, 1.25 mM NaH_2_PO_4_, 26 mM NaHCO_3_, and 10 mM glucose.

Slices were transferred to a recording chamber superfused (2 mL/min) with ACSF at 32~34°C. Slices were visualized with infrared optics using an upright fixed microscope equipped with a ×40 water-immersion lens (FN-S2N, Nikon) and infrared CCD monochrome video camera (IR-1000, DAGE-MTI). The patch pipettes were pulled by a horizontal pipette puller (P-1000; Sutter Instrument) with a resistance of 3~5 MΩ. The recording was performed with the MultiClamp 700B amplifier and 1550 A digitizer (Molecular Device). Series resistance was below 20 MΩ and monitored throughout the experiments.

For spontaneous IPSCs (sIPSCs) recording, PyNs were held at −70 mV in the presence of 20 µM CNQX and 100 µM DL-AP5, with the pipette solution containing: 130 mM KCl, 10 mM HEPES, 0.2 mM EGTA, 1 mM MgCl_2_, 4 mM Mg-ATP, 0.3 mM Na-GTP, and 10 mM phosphocreatine (pH 7.35, 290~295 mOsm). mIPSCs were recorded in the presence of 1 µM TTX.

For spontaneous excitatory postsynaptic currents (sEPSCs) recording, PyNs were held at −70 mV in the presence of 20 µM bicuculline, with the pipette solution containing: 125 mM K-gluconate, 5 mM KCl, 10 mM HEPES, 0.2 mM EGTA, 1 mM MgCl_2_, 4 mM Mg-ATP, 0.3 mM Na-GTP, and 10 mM phosphocreatine (pH 7.3, 290~295 mOsm). miniature EPSCs (mEPSCs) were recorded in the presence of 1 µM TTX.

### Lentivirus preparation

Recombinant lentiviruses were produced by transfected with four plasmids, including pUltra-EGFP-P2A-flag-RLD or pUltra, cmv-VSV-G, RSV-rev, and MDL, to HEK293T cells (at a confluent ~40–50%) by using PEI. Cmv-VSV-G, RSV-rev, and MDL encode the essential elements for packaging the viral particles. 3 days later after transfection, collect all the supernatant and replace it with a fresh culture medium. Collect the second supernatant after culturing for 3 days. All the supernatant are centrifuged at 2000 rpm for 3 min to remove cell debris before filtering them through a 0.45 µm filter. Filtered media was applied to ultracentrifuge 20,000 rpm at 4°C for 2 h. Pellets in the bottom were resuspended with 0.7 mL PBS each tube by vortex for 2~3 min. All resuspended virus was transferred into a new tube and 20% sucrose buffer was added to full the whole tube. Tubes were spin at 20,000 rpm at 4°C for 2 h. The final pellet was resuspended with 80 µL PBS by vortexing for 1 min and pipetting. Aliquot all and store at −80°C.

### Stereotaxic injections

Anesthetized P30 mice were secured in a stereotaxic apparatus. Holes were drilled into the skull and purified protein (1 µg/µL, 1 µL, one side) were microinjected into the dorsal hippocampus using a glass pipette with a fine tip at a rate of 100 nL/min (coordinates: AP −1.70 mm, ML ±1.10 mm and DV +1.10 mm). After injection, pipettes were left in place for 5 min to allow for diffusion of injected protein before being slowly withdrawn. Mice were sacrificed 2 days later after injection. Injection locations were validated in each mouse after experiments.

### Statistical analysis

Data were analyzed with GraphPad Prism version 7.0. for the difference between two groups, Student’s *t* test was used. For three or more groups, one-way analysis of variance was used for analysis. Data were expressed as mean ± SEM. All studies were two-tailed and a significant difference was accepted when the *p* value was <0.05.

## Results

### Induction of inhibitory postsynaptic differentiation by HEK293T cells expressing ErbB4

**Fig. 1:**
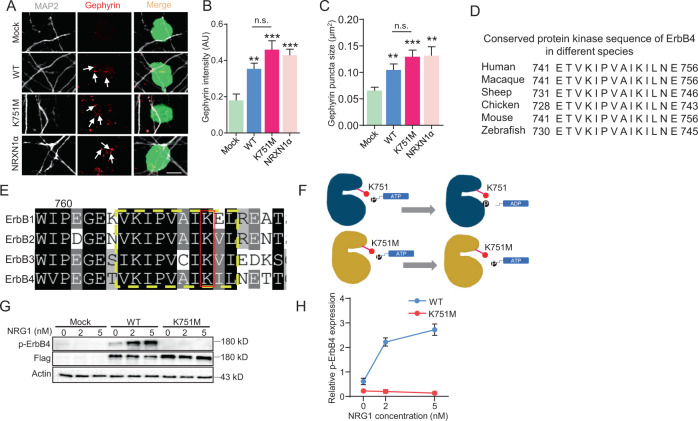
ErbB4 induces inhibitory postsynaptic gephyrin puncta. **A**–**C** ErbB4-transfected HEK293T cells could induce gephyrin puncta. HEK293T cells expressing ErbB4-WT, K751M, NRXN1α, or empty vector (Mock) were co-cultured with hippocampal neurons (DIV9~10) and stained for MAP2 and gephyrin. Arrows indicate GABA_A_R α1 puncta contacting with dendrites. Scale bar, 10 μm (**A**). Quantitative analysis of the intensity (**B**) and size (**C**) of gephyrin puncta in **A**. *n* = 20 cells for Mock, *n* = 21 cells for WT, *n* = 29 cells for K751M, and *n* = 29 cells for NRXN1α. Data were shown as mean ± SEM; **p < 0.01, ***p < 0.001, n.s. *p* > 0.05. Student’s *t* test. **D**–**E** The kinase domain of ErbB4 in different species (**D**) and K751 in ErbB family (**E**) are highly conserved. **F** Schematic illustration for kinase-dead of K751M mutant. **G**–**H** The kinase activity of K751M was completely lost. HEK293T cells were transfected with Flag-tagged ErbB4-WT, K751M, or empty vector (Mock) and treated with different concentrations of NRG1 for 15 min. The cells were lysed and probed with indicated antibodies (**G**). The relative intensities of phosphorylated ErbB4 (p-ErbB4, Y1284) to Flag-ErbB4 from three independent experiments were quantified (**H**). Data were shown as mean ± SEM.

ErbB4 expression is almost exclusively to INs, specifically parvalbuminn+ (PV+) INs [16, 17, 20, 22, 37–42]. Genetic deletion of ErbB4 in PV+ INs decreases the number of inhibitory synapses onto cortical PyNs, indicating that ErbB4 may promote inhibitory synapse formation [16, 26]. To test this possibility, we transfected HEK293T cells with vectors expressing wild-type (WT) ErbB4, NRXN1α, or control empty vector (Mock), respectively. The transfected HEK293T cells were co-cultured with primary hippocampal neurons for 48 h and stained for gephyrin, a postsynaptic marker of inhibitory synapses, along with MAP2 to label dendrites. Interestingly, we found ErbB4-WT was able to induce gephyrin puncta as efficiently as NRXN1α, a presynaptic cell adhesion molecule that has been implicated in inhibitory synapse formation (Fig. 1A). Similar to NRXN1α, both gephyrin puncta intensity (Fig. 1B) and size (Fig. 1C) were increased in ErbB4-WT-expressing cells, comparing with Mock group. These results suggest the role of ErbB4 in inducing inhibitory postsynaptic differentiation.

Considering that ErbB4 is a receptor tyrosine kinase, we determined whether this effect requires its kinase activity. We mutated lysine (K) 751, a conserved amino-acid residue in ErbB (Fig. 1D, E), and EGFR family critical to ATP binding, to methionine (M), which was shown to eliminate the kinase activity of EGFR (Fig. 1F) [43]. When transfected into HEK293T cells, the tyrosine-phosphorylation of ErbB4-WT (p-ErbB4, Y1284) was increased upon NRG1 stimulation in a concentration-dependent manner. However, p-ErbB4 was undetectable in HEK293T cells expressing K751M, indicating that K751M is a completely kinase-dead mutant (Fig.1G, H).

Absorbingly, K751M-expressing cells were also able to induce gephyrin puncta in co-cultured neurons (Fig. 1A). Quantitatively, there were no differences in the intensity (Fig. 1B) and size (Fig. 1C) of gephyrin puncta between WT and K751M. Further, we confirmed these effects by staining for α1 subunit of GABA_A_R, which was localized to the inhibitory postsynaptic membrane. As shown in Fig. S1, increased intensity and size of GABA_A_R α1 puncta were observed in WT and K751M-expressing HEK293T cells. Taken together, these results indicate that ErbB4 could induce postsynaptic differentiation in a kinase-independent manner and suggest that ErbB4 may function as a cell adhesion molecule.

### Abolish of ErbB4 kinase activity in K751M mouse brain

**Fig. 2:**
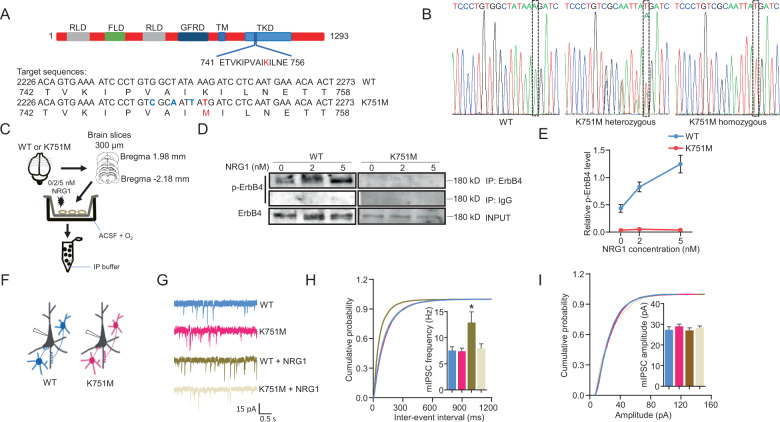
ErbB4 kinase activity is lost in K751M mice. **A** Illustration of the target sequence for K751M mouse generation via CRISPR-Cas9 strategy. **B** The sequencing results of K751M mice genotyping. As indicated in the dashed box: for WT, only one peak of nucleotide A was observed; for K751M heterozygous, both peaks of nucleotide A and T were overlapped; for K751M homozygous, only one peak of nucleotide T was observed. **C**–**E** Abolish of ErbB4 kinase activity in K751M brain slices. Diagram showing the procedure of brain slice preparation and NRG1 treatment (**C**). Adult male mice were anesthetized with isoflurane and killed by decapitation. Brains were quickly removed to the ice-cold oxygenated cutting solution, and incubated with oxygenated ACSF solution. Different concentrations of NRG1 were added to incubate for 15 min. Then, the slices were collected for IP with anti-ErbB4 antibody or IgG. The precipitated complex and inputs were probed with indicated antibodies (**D**). The relative intensities of p-ErbB4 to ErbB4 in **D** from three independent experiments were quantified (**E**). *n* = 6 mice for each genotype. **F**–**G** Abolish of NRG1-induced GABAergic transmission in K751M mice. Schematics of whole-cell patch-clamp recordings of PyNs in CA1 from WT and K751M mice (**F**). Representative traces of mIPSCs (**G**). Scale bars, 15 pA, 0.5 s. Cumulative probability plots and histogram summary of mIPSCs interevent intervals (**H**) and amplitude (**I**) in **G**. *n* = 10 neurons from three WT mice; *n* = 9 neurons from three K751M mice; *n* = 10 neurons from three WT mice treated with NRG1; *n* = 12 neurons from three K751M mice treated with NRG1. Data were shown as mean ± SEM. *p < 0.05. One-way ANOVA.

To test the hypothesis in vivo, we generated K751M knock-in mice by the CRISPR-Cas9 strategy (Fig. 2A). Genomic DNA sequencing indicated correct mutation in the *ErbB4* gene (Fig. 2B). Unlike Null mice that die at E11 because of defective heart development [44], K751M homozygous mice were viable at birth and fertile, without apparent change in life expectancy (Fig. S2A-2B). To determine whether K751M is true “kinase-dead”, brain slices were prepared from WT or K751M homozygous mice, and treated with NRG1 at different concentrations in ACSF for 15 min and lysed (Fig. 2C). The lysates were subjected to IP with anti-ErbB4 antibody and probed for p-ErbB4. As shown in Fig. 2D, E, p-ErbB4 was increased with increasing concentrations of NRG1 in WT mice, while p-ErbB4 was not detected in the complex precipitated with IgG, indicating the specificity of anti-p-ErbB4 antibody. Remarkably, p-ErbB4 was not detectable in slices from K751M homozygous mice (Fig. 2D, E). These results suggest that K751M could not be activated in vivo.

NRG1 is known to increase the frequency of miniature inhibitory postsynaptic currents (mIPSCs) in the hippocampus in a manner dependent on ErbB4 kinase activity [23, 37, 45]. Next, we determined whether K751M alters mIPSCs. Hippocampal CA1 PyNs were recorded in the presence of tetrodotoxin (TTX) to block action potentials and CNQX plus DL-AP5 to block glutamatergic transmission (Fig. 2F). As shown in Fig. 2G–I, NRG1 treatment (5 nM, 15 min) increased the mIPSCs frequency, but not amplitude in WT slices, in agreement with earlier reports [23]. However, this effect was occluded in K751M mice (Fig. 2H). These data support NRG1’s ability to increase mIPSCs frequency is impaired in K751M mice, alluding to a model where ErbB4 kinase activity is needed for inhibitory transmission.

### Normal gross anatomy and number of INs in K751M mouse brain

Although NRG1 was unable to increase mIPSCs frequency in K751M slices, baseline levels of both mIPSCs frequency and amplitude were equivalent to WT animals (Fig. 2H, I), suggesting that baseline levels of GABAergic transmission are not altered in K751M mice. Nevertheless, ErbB4 has been implicated in neural development, specifically due to its kinase activity is believed to be essential for INs distribution in the cortex and hippocampus [46, 47]. To determine whether ErbB4 kinase inactivation impairs brain structure, brain sections were harvested for Nissl staining. As shown in Fig. S2C, gross brain morphology was normal in K751M homozygous mice as well as Null in alignment with previous studies [48]. To determine whether K751M mutation disturbs INs migration, K751M or Null mice were crossed with *GAD67*::GFP mice that express GFP in GABAergic INs. Brain sections were prepared and co-stained with GFP and NeuN antibodies. In keeping with previous reports [46, 49, 50], the number of GAD67+ INs was reduced by ~30% in Null mice (Fig. S3A–D). In contrast, the numbers of GAD67+ INs in the somatosensory cortex and hippocampus were similar between K751M homozygous and WT mice (Fig. S3A–D). As previous papers reported, ~70–80% PV+ INs in the somatosensory cortex and hippocampus are ErbB4+ [51] and the number of PV+ INs was reduced in Null mice [46, 50, 52]. We stained brain sections with PV and NeuN antibodies and observed a reduction in PV+ INs in the somatosensory cortex and hippocampus of Null mice (Fig. S3E–H). Nonetheless, the number of PV+ INs was also similar between K751M homozygous and WT mice. These results indicate that ErbB4 kinase is dispensable for INs to populate the cortex and hippocampus, suggesting that ErbB4 might regulate INs development independent of its kinase activity.

### A comparable number of inhibitory synapses onto PyNs in K751M mice

**Fig. 3:**
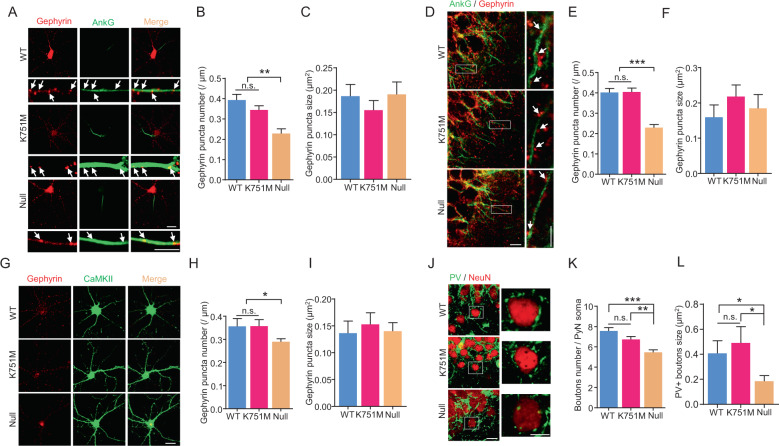
ErbB4 protein, but not its kinase activity, is important for inhibitory synapse formation. **A**–**C** AIS gephyrin puncta number was decreased in primary Null hippocampal neurons, but not in K751M neurons. Representative images of gephyrin+ inhibitory synapses onto AnkG+ AIS of cultured hippocampal PyNs (**A**). Arrows indicate gephyrin puncta contacting with AIS. Scale bar, 20 μm for lower magnification images and 10 μm for enlarged magnification images. Quantitative analysis of AIS gephyrin puncta number (**B**) and size (**C**) in **A**. *n* = 39 neurons for WT, *n* = 35 neurons for K751M, and *n* = 20 neurons for Null. **D**–**F** AIS gephyrin puncta number was decreased in the hippocampus of Null mice, but not K751M mice. Representative images of gephyrin+ inhibitory synapses onto AnkG+ AIS of CA1 PyNs in brain slices. Boxed areas were enlarged in the respective right images (**D**). Arrows indicate gephyrin puncta contacting with AIS. Scale bar, 10 μm for lower magnification images and 5 μm for enlarged images of boxed areas. Quantitative analysis of AIS gephyrin puncta number (**E**) and size (**F**) in **D**. *n* = 28 neurons from four WT, *n* = 28 neurons from four K751M, and *n* = 28 neurons from four Null mice. **G**–**I** Perisomatic gephyrin puncta number was decreased in primary Null hippocampal neurons, but not in K751M neurons. Representative images of gephyrin+ inhibitory synapses onto perisoma of cultured hippocampal PyNs (**G**). Scale bar, 20 μm. Quantitative analysis of AIS gephyrin puncta number (**H**) and size (**I**) in **G**. *n* = 20 neurons for WT, *n* = 25 neurons for K751M, and *n* = 25 neurons for Null. **J**–**L** Perisomatic gephyrin puncta number was decreased in the hippocampus of Null mice, but not K751M mice. Representative images of gephyrin+ inhibitory synapses onto perisoma of CA1 PyNs in brain slices. Boxed areas were enlarged in the respective right images (**J**). Scale bar, 10 μm for lower magnification images and 5 μm for enlarged images of boxed areas. Quantitative analysis of perisomatic gephyrin puncta number (**K**) and size (**L**) in **D**. *n* = 30 neurons from four WT mice, *n* = 35 neurons from four K751M mice, and *n* = 32 neurons from four Null mice. Data were shown as mean ± SEM, *p < 0.05, **p < 0.01, ***p < 0.001, n.s. *p* > 0.05. One-way ANOVA.

PV-expressing basket cells form perisomatic synapses with PyNs, whereas chandelier cells form inhibitory synapses at axon initial segments (AIS) of PyNs [53]. ErbB4 is expressed in both basket and chandelier cells [16, 26] and has been shown necessary for the formation of these inhibitory synapses [16, 22, 26, 39, 49]. To determine whether ErbB4 kinase activity is required for inhibitory synapse formation in vitro, hippocampal neurons were cultured from E18.5 embryos, fixed, and stained for gephyrin, Ankyrin-G (AnkG, an AIS marker), or CaMKII that labeled PyNs somas at DIV12. As shown in Fig. 3A–C, G–I, both AIS and perisomatic gephyrin puncta numbers were decreased in cultured neurons from Null mice, which is consistent with previous reports [16, 49]. Surprisingly, similar gephyrin puncta numbers were observed in cultured neurons from K751M and WT mice (Fig. 3B, H). Moreover, the sizes of gephyrin puncta were comparable among WT, K751M, and Null mice (Fig. 3C, I). Similarly, GABA_A_R α1 and AnkG co-staining also showed AIS GABA_A_R α1 puncta number was not changed in K751M mice but decreased in Null mice (Fig. S4A, B).

To validate these phenotypes in vivo, brain sections were prepared from these mice at P35, then stained for gephyrin and AnkG. AIS inhibitory synapse number onto PyNs was decreased in Null mice, which is similar to ErbB4 conditional knockout mice in the previous report [16], but not in K751M mice (Fig. 3D, E). Next, we co-stained PV and NeuN to examine perisomatic inhibitory synapses onto PyNs in vivo. Similarly, we found that the perisomatic inhibitory synapse number was not altered in K751M mice, but decreased in Null mice (Fig. 3J, K). And the size of perisomatic inhibitory synapses was reduced in Null mice, but not in K751M (Fig. 3J, L), whereas the size of AIS inhibitory synapses was similar among these three mice (Fig. 3D, F). Decreased PV+ INs in Null mice may contribute to this observation. These results indicate ErbB4 kinase activity is dispensable for inhibitory synapse formation in vitro and in vivo.

To detect the expression of different inhibitory synaptic proteins in different brain regions of K751M mice. We harvested tissues from the whole brain, cortex (prelimbic cortex, PrL), and hippocampus. We found that the expressions of synaptophysin (SYN), vesicular GABA transporter (VGAT, an inhibitory presynaptic marker), gephyrin, and glutamate decarboxylase 67 (GAD67, an INs marker) were not changed in K751M mice, but decreased in Null mice (Fig. S4C, D). Taken together, these results suggest that ErbB4, but not its kinase activity, is important for inhibitory synapse formation onto PyNs in CA1.

**Fig. 4:**
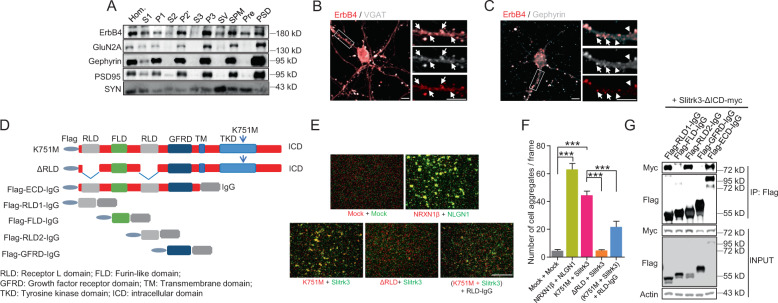
ErbB4 interacts transcellularly with Slitrk3. **A** ErbB4 was distributed to the presynaptic and postsynaptic areas. Subcellular fractions of the WT brains were blotted with anti-ErbB4, anti-GluN2A (a postsynaptic glutamate receptor), anti-gephyrin (an inhibitory postsynaptic marker), anti-PSD95 (a PSD marker), and anti-synaptophysin (SYN, a presynaptic marker). Hom, homogenate; S1, supernatant; P1, pelleted nuclear fraction; P2, crude synaptosomal fraction; P2’, washed crude synaptosomal fraction; S3, crude synaptic vesicle fraction; P3, lysed synaptosomal membrane fraction; SV, synaptic vesicle fraction; SPM, synaptosomal plasma membrane; Pre, presynaptic fraction; PSD, postsynaptic density. **B**, **C** Colocalization of ErbB4 with VGAT (**B**) or gephyrin (**C**). Primary hippocampal neurons at DIV10 were co-stained with ErbB4 and VGAT or gephyrin. Boxed areas were enlarged in the respective right images. Arrows indicate the co-localized ErbB4 with VGAT or gephyrin, and arrowheads indicate ErbB4 puncta alone. Scale bar, 20 μm for lower magnification images and 10 μm for enlarged images of boxed areas. **D** Schematic illustration of constructs with different ErbB4 domain structures. **E**, **F** In trans ErbB4–Slitrk3 interaction required the RLD of ErbB4. Representative images of cell aggregation assay (**E**). Scale bar, 1 mm. HEK392T cells co-transfected with DsRed and K751M, ΔRLD, NRXN1β or Mock mixed with HEK392T cells co-transfected with EGFP and Slitrk3, NLGN1 or Mock. In K751M + Slitrk3 + RLD-IgG group, recombinant RLD-IgG protein was added to the mixture of K751M cells and Slitrk3 cells. Quantitative analysis of cell aggregates in **E** (**F**). Data were from three independent experiments and shown as mean ± SEM. ***p < 0.001. One-way ANOVA. **G** ErbB4 interacted with Slitrk3 through RLD domain. HEK293T cells were co-transfected with myc-tagged Slitr3-ΔICD plus Flag-tagged different ECD domains of ErbB4 and subjected to co-IP with Flag-beads.

### Trans interaction between ErbB4 and Slitrk3

ErbB4 has been reported to distribute at the postsynaptic region [54, 55]. To determine whether it is also present in the presynaptic compartment, whole mouse brains were isolated and fractionated by discontinuous sucrose density centrifugation. As shown in Fig. 4A, ErbB4 was found enriched in the PSD fraction where gephyrin, PSD95, or GluN2A were enriched, confirming earlier reports [54, 55]. It was also present in the presynaptic membrane fraction where SYN was enriched, indicating that ErbB4 is present in presynaptic axon terminals. Furthermore, our immunostaining results from cultured hippocampal neurons showed the colocalization of ErbB4 with VGAT (Fig. 4B) or gephyrin (Fig. 4C). ErbB4 protein, but not its activity, was required for inhibitory synapse formation onto PyNs (Fig. 3). ErbB4, as a single transmembrane protein (Fig. 4D), might be a cell adhesion molecule to promote synapse formation. We reviewed all reported cell adhesion molecules that are localized to the inhibitory postsynaptic area. Among them, NLGN2~4 and Slitrk3 could induce inhibitory presynaptic differentiation [13, 15, 56–58]. Co-IP experiments suggest that ErbB4 did not interact with NLGN2~4 (data not shown). Slitrk3 is a single transmembrane protein at the inhibitory postsynaptic area and is critical for inhibitory synapse formation [15]. To determine whether there is a transcellular interaction between ErbB4 and Slitrk3, a cell aggregation assay was performed. As a positive control, NRXN1β-expressing HEK293T cells (RFP+ cells) formed aggregates with NLGN1-expressing HEK293T cells (GFP+ cells) (Fig. 4E, F), which is consistent with the previous report [8]. Nonetheless, cells transfected with empty vectors (Mock) did not form aggregates (Fig. 4E, F). Interestingly, K751M-expressing cells aggregated with Slitrk3-expressing cells (Fig. 4E, F), indicating in trans interaction of ErbB4 to Slitrk3. Within the extracellular domain (ECD) of ErbB4, there are two receptor L domains (RLDs), a Furin-like domain (FLD) and a growth factor receptor domain (GFRD) (Fig. 4D). To map the domain of ErbB4 binding with Slitrk3, different ECD domains of ErbB4 were co-transfected with Slitrk3 without intracellular domain (Slitrk3-ΔICD) into HEK293T cells for co-IP. As shown in Fig. 4G, Slitrk3-ΔICD co-IPed with RLD1 or RLD2, suggesting RLDs of ErbB4 interact with Slitrk3. Next, we constructed K751M without RLDs (ΔRLD) (Fig. 4D) for cell aggregation assay. ΔRLD-expressing cells did not form aggregates with Slitrk3-expressing cells (Fig. 4E, F). These results indicate a requirement of RLD for ErbB4–Slitrk3 transcellular interaction. In agreement, the reduced aggregate formation was observed when recombinant RLD-IgG protein was added into the mixtures of K751M-expressing and Slitrk3-expressing cells (Fig. 4E, F). Taken together, these results suggest ErbB4, as a cell adhesion molecule, interacts transcellularly with Slitrk3.

### Impaired inhibitory synapse formation upon disruption of the ErbB4–Slitrk3 interaction

**Fig. 5:**
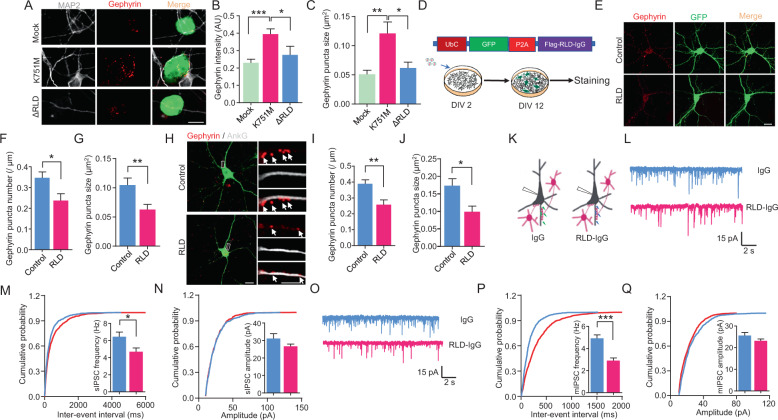
ErbB4–Slitrk3 interaction is important for inhibitory synapse formation and transmission. **A**–**C** ErbB4-ΔRLD-expressing HEK293T cells could not induce gephyrin puncta. HEK293T cells expressing K751M, ΔRLD, or empty vector (Mock) were co-cultured with hippocampal neurons (DIV9) and stained for MAP2 and gephyrin. Scale bar, 10 μm (**A**). Quantitative analysis of the intensity (**B**) and size (**C**) of gephyrin puncta in **A**. *n* = 30 cells for Mock, *n* = 19 cells for K751M, and *n* = 23 cells for ΔRLD. **D**–**G** Perisomatic gephyrin puncta number and size were decreased in primary hippocampal neurons infected with RLD-expressing virus. Schematic of lentivirus vector expressing Flag-RLD-IgG and experimental timeline (**D**). Hippocampal neurons at DIV2 were infected with the virus expressing RLD or empty vector (Control) for 10 days and then fixed and stained with gephyrin and GFP antibodies. Representative images of gephyrin+ inhibitory synapses onto perisoma of cultured PyNs (**E**). Scale bar, 20 μm. Quantitative analysis of perisomatic gephyrin puncta number (**F**) and size (**G**) in **E**. *n* = 30 neurons for Control, *n* = 30 neurons for RLD. **H**–**J** AIS gephyrin puncta number and size were decreased in primary hippocampal neurons infected with RLD-expressing virus. Representative images of gephyrin+ AIS inhibitory synapses of cultured PyNs. Boxed areas were enlarged in the respective right images (**H**). Scale bar, 20 μm for lower magnification images and 10 μm for enlarged images of boxed areas. Quantitative analysis of perisomatic gephyrin puncta number (**I**) and size (**J**) in **H**. *n* = 24 neurons for Control, *n* = 24 neurons for RLD. **K**–**Q** RLD injection decreased inhibitory synaptic transmission in hippocampal CA1 PyNs. Schematics of whole-cell patch-clamp recordings of PyNs in CA1 from IgG or RLD-IgG-injected K751M mice (**K**). Representative traces of sIPSCs (**L**) and mIPSCs (**O**). Scale bars, 15 pA, 2 s. Cumulative probability plots and histogram summary of sIPSCs and mIPSCs interevent intervals (**M**, **P**), as well as amplitudes (**N**, **Q**). *n* = 10 neurons from three IgG-injected mice and *n* = 9 neurons from three RLD-IgG-injected mice. Data were shown as mean ± SEM. *p < 0.05, **p < 0.01, ***p < 0.001. Student’s *t* test.

To determine whether the ErbB–Slitrk3 interaction is important for inhibitory synapse formation, we repeated the artificial synapse formation assay by co-culturing ErbB4-ΔRLD-expressing HEK293T cells with DIV9 hippocampal neurons. As shown in Fig. 5A–C and Fig. S5A–C, both the intensity and puncta size of gephyrin or GABA_A_R α1 on contacting dendrites were reduced in ErbB4-ΔRLD-expressing cells, comparing with those in K751M-expressing cells. These results suggest an essential role of the ErbB4–Slitrk3 interaction in postsynaptic induction. Next, we constructed a secretable RLD-expressing vector and packaged it into lentivirus (Fig. 5D). Hippocampal neurons at DIV2 were infected with the virus expressing RLD or empty vector (Control) for 10 days, and then fixed and stained with AnkG, gephyrin, or GABA_A_R α1 antibodies. Compared with the Control, RLD decreased the puncta numbers of gephyrin or GABA_A_R α1 in the perisomatic areas (Fig. 5E, F and Fig. S5D, E) and AIS (Fig. 5H, I and Fig. S5G, H) of PyNs. Interestingly, the puncta sizes of gephyrin or GABA_A_R α1 in the perisomatic areas (Fig. 5G and Fig. S5F) and AIS (Fig. 5J and Fig. S5I) were also reduced upon RLD overexpression, which might be resulted from blocking other Slitrk3 pathways. These results confirmed the induction role of ErbB4–Slitrk3 interaction in inhibitory synapse formation.

Finally, we explored the consequence of disrupting ErbB4–Slitrk3 interaction in vivo. Recombinant RLD-IgG protein or IgG alone was purified from the conditional medium of transfected HEK293T cells with protein A/G-immobilized beads and stereotactically injected into the hippocampal CA1 of K751M homozygous mice. Hippocampal slices were isolated 2 days later after surgery and CA1 PyNs were recorded in whole-cell patch-clamp configuration (Fig. 5K). As shown in Fig. 5L–Q, the spontaneous IPSCs (sIPSCs) frequency of RLD-IgG-injected mice was decreased compared with IgG-injected mice, but the amplitude was not changed. Consistent with sIPSCs, the mIPSCs frequency (Fig. 5O, P), but not amplitude (Fig. 5Q), was also reduced in RLD-IgG-injected mice. Moreover, frequencies and amplitudes of sEPSCs and mEPSCs were not altered in RLD-IgG-injected mice (Fig. S6A–F), suggesting a specific effect of ErbB4–Slitrk3 interaction in inhibitory synaptic transmission. In summary, these data are consistent with a model that ErbB4 in trans interacts with Slitrk3 to promote the formation of inhibitory synapses onto PyNs.

## Discussion

In this study, we provide evidence for a novel mechanism of ErbB4 in inhibitory synapse formation. First, overexpressing ErbB4-WT or its kinase-dead mutation (K751M) in HEK293T cells induced gephyrin or GABA_A_R α1 puncta in co-cultured primary hippocampal neurons. Moreover, in K751M knock-in homozygous mice, the numbers of INs and inhibitory synapses onto PyNs were not impaired. Second, we identified Slitrk3, a transmembrane postsynaptic protein at inhibitory synapses, as an in trans binding partner for ErbB4. Through cell aggregation and co-IP assays, we mapped the RLD domain of ErbB4 for its interaction with Slitrk3. Finally, overexpressing secretable RLD in primary hippocampal neurons to disrupt ErbB4–Slitrk3 interaction decreased inhibitory synapses onto PyNs in the AIS and perisomatic areas. Injecting recombinant RLD protein in the hippocampal CA1 of K751M mice decreased the frequencies of sIPSCs and mIPSCs. Taken together, these observations support a working hypothesis that ErbB4 may function as a cell adhesion molecule to promote inhibitory synapse formation via in trans interacting with Slitrk3.

ErbB4 almost expresses in INs during development and maturation [16, 17, 23, 26, 38, 47, 51, 59]. And it is essential for the assembly of the GABAergic circuitry, such as INs migration from median ganglionic eminence (MGE) and synapse formation [16, 18, 19, 22, 26]. ErbB4-Null mice display fewer numbers of INs and PV+ INs (Fig. S3) [46, 49, 50]. Nevertheless, how ErbB4 promotes INs migration is controversial. Nuria Flames et al. [46] thought NRG1 acted as short- and long-range attractants for INs migration by NRG1/ErbB4 signaling pathway. By in vitro focal electroporation of dominant-negative ErbB4 (dnErbB4, the ErbB4-ΔICD mutant) into the MGE of coronal slice cultures, they found loss of ErbB4 function impaired INs migration to the cortex [46]. However, another study also used the same assay and found ErbB4-ΔICD expressing MGE cells could migrate to the cortex, which was similar to the control empty vector expressing cells. And surprisingly, ErbB4-overexpressing MGE cells also exhibited migration defects [60]. However, In vitro overexpression system might induce artificial phenotypes that are controversial among different experiment systems. Here, our K751M mice were the knock-in mice with a single mutation to inactivate ErbB4 kinase activity in vivo (Fig. 2). The numbers and distributions of INs in the cortex and hippocampus of K751M mice were not changed (Fig. S3). Therefore, we proposed that the cell adhesion function of ErbB4 might be important for INs migration, which could be verified furtherly if RLD deletion mice were available.

Synapse formation is a key step for the assembly of neuronal circuitry, and ErbB4 is critical for synapse formation. At the subcellular level, ErbB4 is not only localized to the postsynaptic area of excitatory and inhibitory synapses in GABAergic INs [54, 55] but also can be found in the axonal terminal of INs [16, 26]. Similarly, we also showed that ErbB4 was detected in both presynaptic and postsynaptic fractions in the PSD fractionation assay (Fig. 4A) in vivo and co-localized with VGAT (Fig. 4B) or gephyrin (Fig. 4C) in vitro. ErbB4 has been reported to promote the synaptogenesis of excitatory synapses on INs, as well as inhibitory synapses onto PyNs [20, 22, 61, 62]. Interestingly, a previous report indicates that the inhibitory synapse onto INs shows no change in the presence of NRG1 [22], which suggests it may be an ErbB4 kinase activity-independent manner. We also found that the role of ErbB4 in the formation of inhibitory synapses onto PyNs was independent of its kinase activity (Fig. 3 and Fig. S4A, B). Overexpressing ErbB4 in HEK293T cells can induce gephyrin or GABA_A_R α1 puncta in co-cultured primary hippocampal neurons (Fig. 1A–C and Fig. S1). Also, the number of inhibitory synapses on AIS and perisomatic areas was reduced in primary cultured hippocampal neurons or brain sections from Null mice, but not in K751M homozygous mice (Fig. 3). Thus, we proposed that ErbB4 might act as a cell adhesion molecule to promote inhibitory synapse formation.

Cell adhesion molecules, such as the NRXNs and NLGNs, NGL, LRRTMs, EphrinBs, and EphBs, are well known for their critical roles in organizing synapse formation and maturation. EphB2, as a transmembrane tyrosine kinase receptor, is demonstrated to be a postsynaptic signal to trigger presynaptic differentiation through ephrin binding in mice and *Xenopus* [10, 63, 64]. Similarly, ErbB4 is also a transmembrane tyrosine kinase receptor and reported to modulate both excitatory and inhibitory synapses formation. Here, for the first time, we reported that ErbB4 was able to induce inhibitory postsynaptic differentiation (Fig. 1A–C and Fig. S1) and promoted inhibitory synapse formation, independent of its kinase activity (Fig. 3). Moreover, these effects were impaired when blocking the ErbB4–Slitrk3 interaction by deletion of ErbB4 RLD or overexpressing RLD (Fig. 5A–J and Fig. S5). These results further suggest the cell adhesion function of ErbB4 in promoting inhibitory synapse formation.

Few studies have been conducted to explore the mechanism of inhibitory synapse formation onto PyNs. Based on previous reports, NLGN2 and Slitrk3 are believed to regulate inhibitory synapse formation [58, 65–68]. Unlike NLGN2, which is well-studied in vitro and in vivo [13, 66, 67, 69, 70], Slitrk3 is less understood. In vitro studies have indicated that Slitrk3 knockdown results in reduced inhibitory synapse density [15]. Slitrk3^−/−^ mice exhibited deficiency in inhibitory synapses number and function, as well as increased seizure susceptibility and spontaneous epileptiform activity, which is similar to ErbB4 conditional knockout mice [68]. Slitrk3 interacts with NLGN2 through its LRR9 domain within ECD, and the density of inhibitory synapses in the CA1 region of Slitrk3^ΔLRR9/ ΔLRR9^ is impaired [58]. Our study found that ErbB4 only interacts with Slitrk3 but not NLGN2 through their ECD domains (Fig. 4E), suggesting that ErbB4 might recruit gephyrin or GABA_A_R α1 puncta into the postsynaptic area through NLGN2-Slitrk3 complex [66].

In summary, our study demonstrated ErbB4 acted as a cell adhesion molecule to promote inhibitory synapse formation. Disruption of the ErbB4–Slitrk3 interaction impairs inhibitory synapse formation and GABAergic transmission (Fig. 5). To further clarify these functions, RLD overexpression or RLD deletion mice could be generated.

## Supplementary information

Supplementary Figure Legends

Supplementary Figure 1

Supplementary Figure 2

Supplementary Figure 3

Supplementary Figure 4

Supplementary Figure 5

Supplementary Figure 6
