# Supplementary material for: ErbB4 promotes inhibitory synapse formation by cell adhesion, independent of its kinase activity
Source: Transl Psychiatry. 2021 Jun 29;11:361. doi: 10.1038/s41398-021-01485-6 (PMC8257755; doi:10.1038/s41398-021-01485-6)
Supplement: Supplementary file 1 — Supplementary Figure Legends [file 41398_2021_1485_MOESM1_ESM.docx]

**Supplementary Figure Legends**

**Fig. S1 ErbB4 induces inhibitory postsynaptic GABA_A_R α1 puncta.**

HEK293T cells expressing ErbB4-WT, K751M, NRXN1α or empty vector (Mock) were co-cultured with hippocampal neurons (DIV9~10) and stained for MAP2 and GABA_A_R α1. Arrows indicate GABA_A_R α1 puncta contacting with dendrites. Scale bar, 10 μm (**A**). Quantitative analysis of the intensity (**B**) and size (**C**) of GABA_A_R α1 puncta in **A**. n = 15 cells for Mock, n = 35 cells for WT, n = 21 cells for K751M and n = 20 cells for NRXN1α. Data were shown as mean ± SEM; *p < 0.05, ***p < 0.001. Student's *t*-test.

**Fig. S2 K751M mice exhibit normal gross brain anatomy and body size.**

**A** Normal body size between WT and K751M male mice at P1 and P60. **B** Normal body weight between WT and K751M mice. Quantitative analysis of body weight for each genotype. Mice weight was measured every 4 weeks and recorded. n = 10 mice for each genotype. **C** Normal brain anatomy in K751M and Null mice. HIP: hippocampus, Nc: neocortex, Th: thalamus, CPu: caudate putamen, Hy: hypothalamus, Am: amygdala. Scale bar, 500 μm.

**Fig. S3 ErbB4 protein, but not kinase activity, is necessary for populating INs in the cortex and hippocampus.**

**A-D** The number of GAD67+ INs in the somatosensory cortex (**A** and **B**) and hippocampus (**C** and **D**) was similar between K751M and WT mice. WT, K751M and Null mice were sacrificed for staining with GFP and NeuN antibodies. Representative images in the somatosensory cortex (**A**) and hippocampus (**C**). Scale bar, 25 μm. CA, cornu Ammonis; DG, dentate gyrus. Quantitative analysis of GAD67+ neuron number in **A** (**B**) and **C** (**D**). n = 27 images from four WT mice, n = 27 images from four K751M mice, n = 33 images from four Null mice. **E-H** The number of PV+ INs in the somatosensory cortex (**E** and **F**) and hippocampus (**G** and **H**) was similar between K751M and WT mice. WT, K751M and Null mice were sacrificed for staining with PV and NeuN antibodies. Representative images in the somatosensory cortex (**E**) and hippocampus (**G**). Scale bar, 25 μm. Quantitative analysis of GAD67+ neuron number in **E** (**F**) and **G** (**H**). n = 22 images from three WT mice, n = 30 images from three K751M mice, n = 34 images from three Null mice. Data were shown as mean ± SEM. *p < 0.05, **p < 0.01, n.s. p > 0.05. One-way ANOVA.

**Fig. S4 Expressions of inhibitory synaptic markers are decreased in Null, but not in K751M mice.**

**A-B** AIS GABA_A_R α1 puncta number was decreased in primary Null hippocampal neurons, but not in K751M neurons. Representative images of GABA_A_R α1+ inhibitory synapses onto AnkG+ AIS of cultured hippocampal PyNs (**A**). Arrows indicate GABA_A_R α1 puncta contacting with AIS. Scale bar, 20 μm for lower magnification images and 10 μm for enlarged magnification images. Quantitative analysis of AIS GABA_A_R α1 puncta number in **A** (**B**). n = 24 neurons for WT, n = 24 neurons for K751M and n = 24 neurons for Null. **C-D** Different synaptic protein expressions in K751M mice. Lysates of the whole brain, cortex (prelimbic cortex, PrL) and hippocampus (Hippo) from WT, K751M and Null mice were subjected to western blotting and probed with indicated antibodies (**C**). Quantitative analysis of relative protein levels in **C** from three independent experiments (**D**). n = 3 mice for each genotype. Data were shown as mean ± SEM. *p < 0.05, **p < 0.01, ***p < 0.001, n.s. p > 0.05. One-way ANOVA.

**Fig. S5. Disrupting Erbb4-Slitrk3 interaction impairs inhibitory synapse formation.**

**A-C** ErbB4-ΔRLD expressing HEK293T cells could not induce GABA_A_R α1 puncta. HEK293T cells expressing K751M, ΔRLD or empty vector (Mock) were co-cultured with hippocampal neurons (DIV9) and stained for MAP2 and GABA_A_R α1. Arrows indicate GABA_A_R α1 puncta contacting with dendrites. Scale bar, 10 μm (**A**). Quantitative analysis of the intensity (**B**) and size (**C**) of GABA_A_R α1 puncta in **A**. n = 24 cells for Mock, n = 23 cells for K751M and n = 24 cells for ΔRLD. **D-F** Perisomatic GABA_A_R α1 puncta number and size were decreased in primary hippocampal neurons infected with RLD-expressing virus. Representative images of GABA_A_R α1+ inhibitory synapses onto perisoma of cultured PyNs (**D**). Scale bar, 20 μm. Quantitative analysis of perisomatic GABA_A_R α1 puncta number (**E**) and size (**F**) in **D**. n = 27 neurons for control and n = 40 neurons for RLD. **G-I** AIS GABA_A_R α1 puncta number and size were decreased in primary hippocampal neurons infected with RLD-expressing virus. Representative images of GABA_A_R α1 AIS inhibitory synapses of cultured PyNs (**H**). Arrows indicate GABA_A_R α1 puncta contacting with AIS. Scale bar, 10 μm. Quantitative analysis of perisomatic GABA_A_R α1 puncta number (**H**) and size (**I**) in **G**. n = 24 neurons for Control and n = 29 neurons for RLD. Data were shown as mean ± SEM. *p < 0.05, **p < 0.01, ***p < 0.001. Student's *t*-test.

**Fig. S6. Disrupting Erbb4-Slitrk3 interaction does not impair excitatory synaptic transmission in hippocampal CA1 PyNs.**

Representative traces of sEPSCs (**A**) and mEPSCs (**D**). Scale bars, 20 pA, 2 s. Cumulative probability plots and histogram summary of sEPSCs and mEPSCs interevent intervals (**B** and **E**), as well as amplitudes (**C** and **F**). For sEPSCs, n = 12 neurons from three mice for each group; for mEPSCs, n = 10 neurons from three mice for each group. Data were shown as mean ± SEM. Student's *t*-test.
