# Supplementary figures and images for: ErbB4 promotes inhibitory synapse formation by cell adhesion, independent of its kinase activity
Source: Transl Psychiatry. 2021 Jun 29;11:361. doi: 10.1038/s41398-021-01485-6 (PMC8257755; doi:10.1038/s41398-021-01485-6)

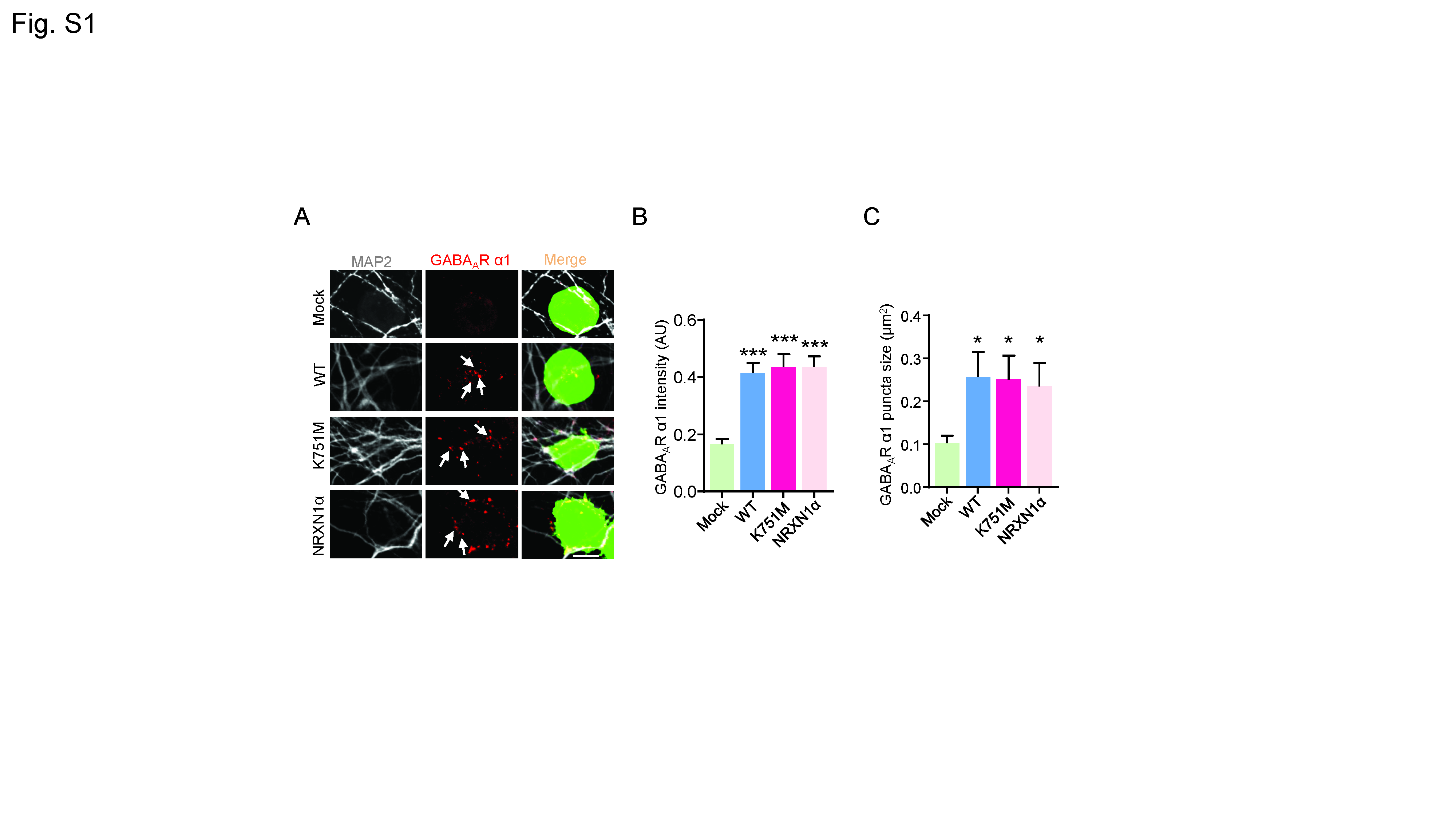

Supplement: Supplementary file 2 — Supplementary Figure 1 [file 41398_2021_1485_MOESM2_ESM.tif]

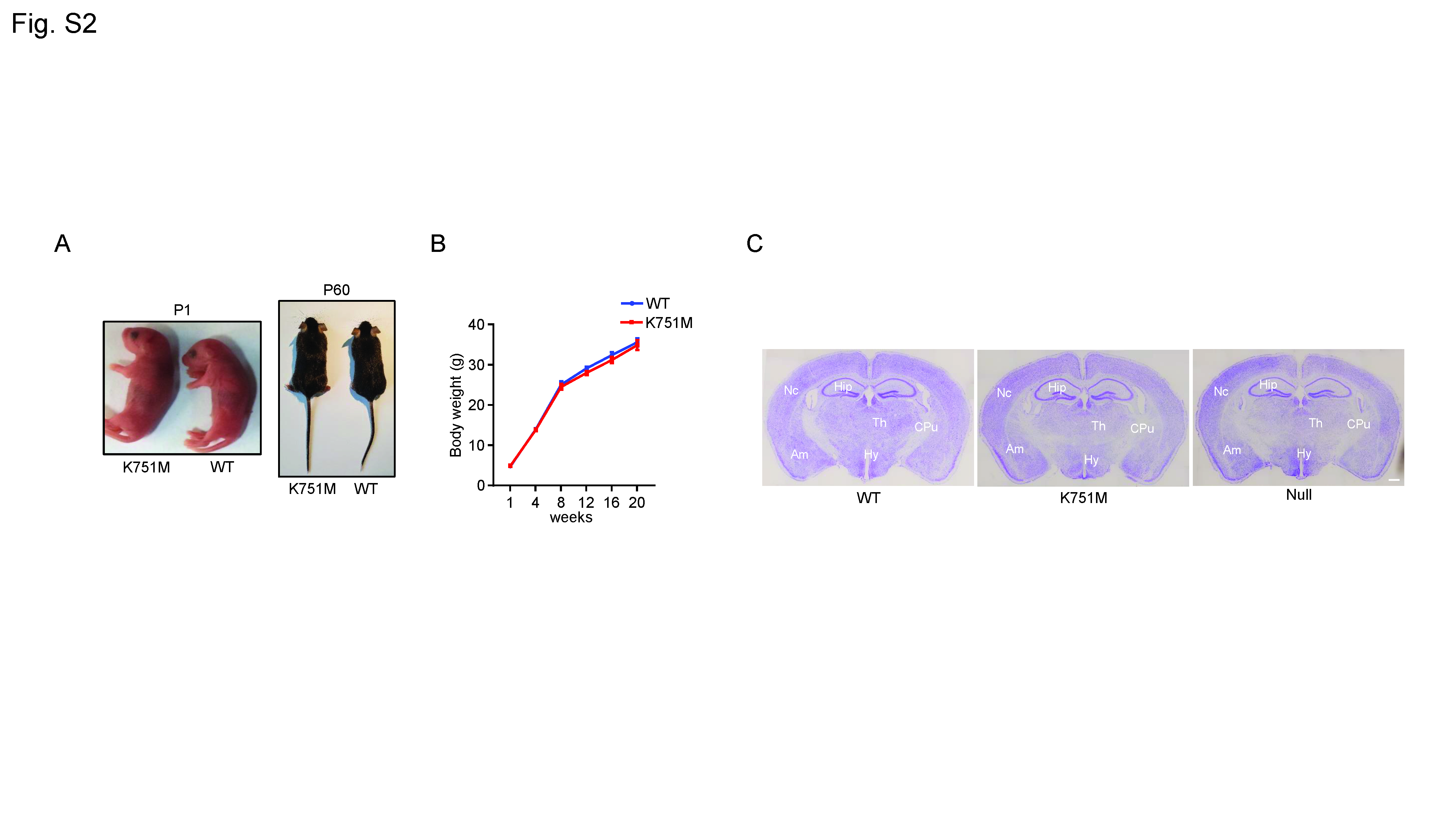

Supplement: Supplementary file 3 — Supplementary Figure 2 [file 41398_2021_1485_MOESM3_ESM.tif]

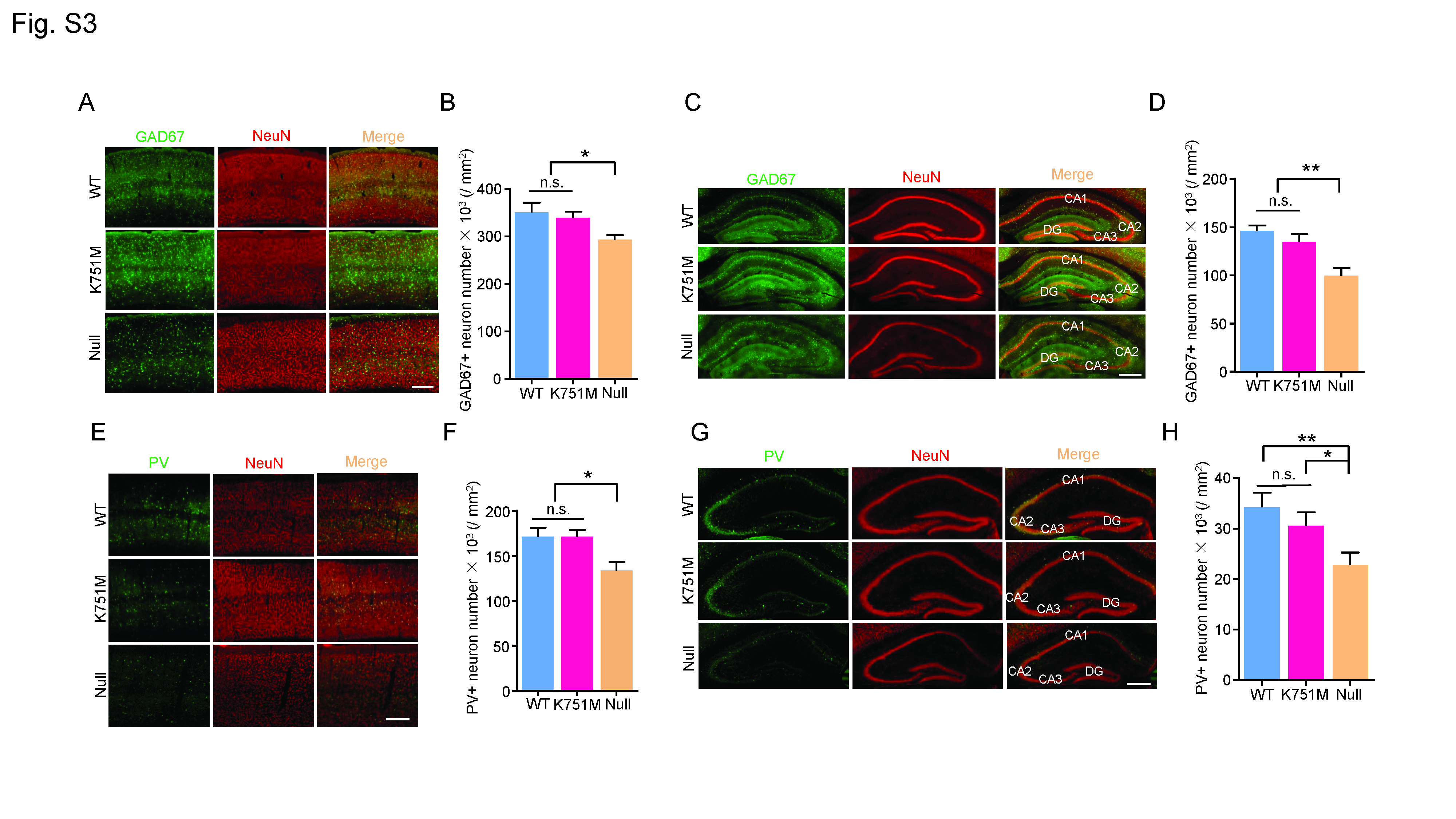

Supplement: Supplementary file 4 — Supplementary Figure 3 [file 41398_2021_1485_MOESM4_ESM.tif]

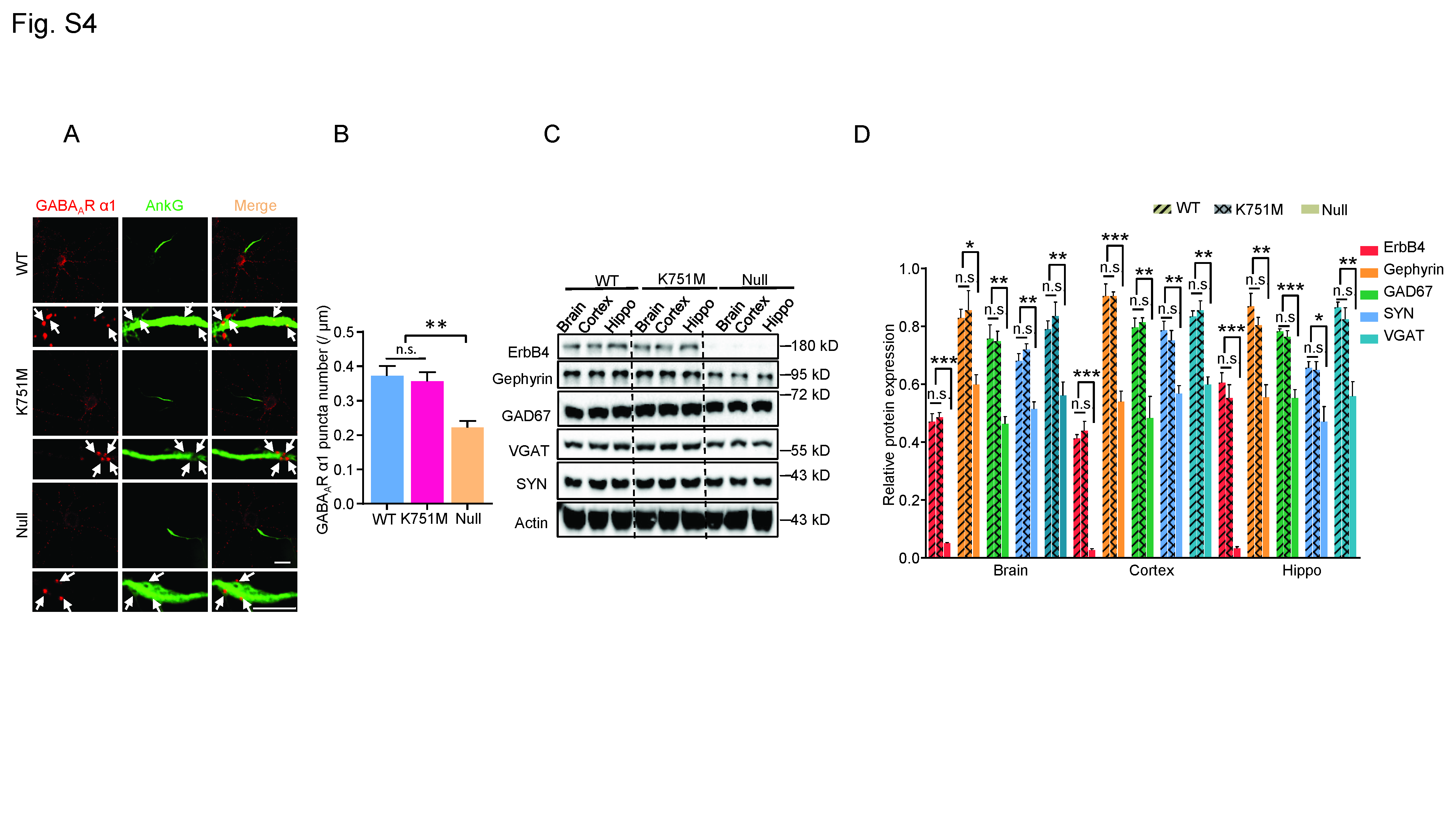

Supplement: Supplementary file 5 — Supplementary Figure 4 [file 41398_2021_1485_MOESM5_ESM.tif]

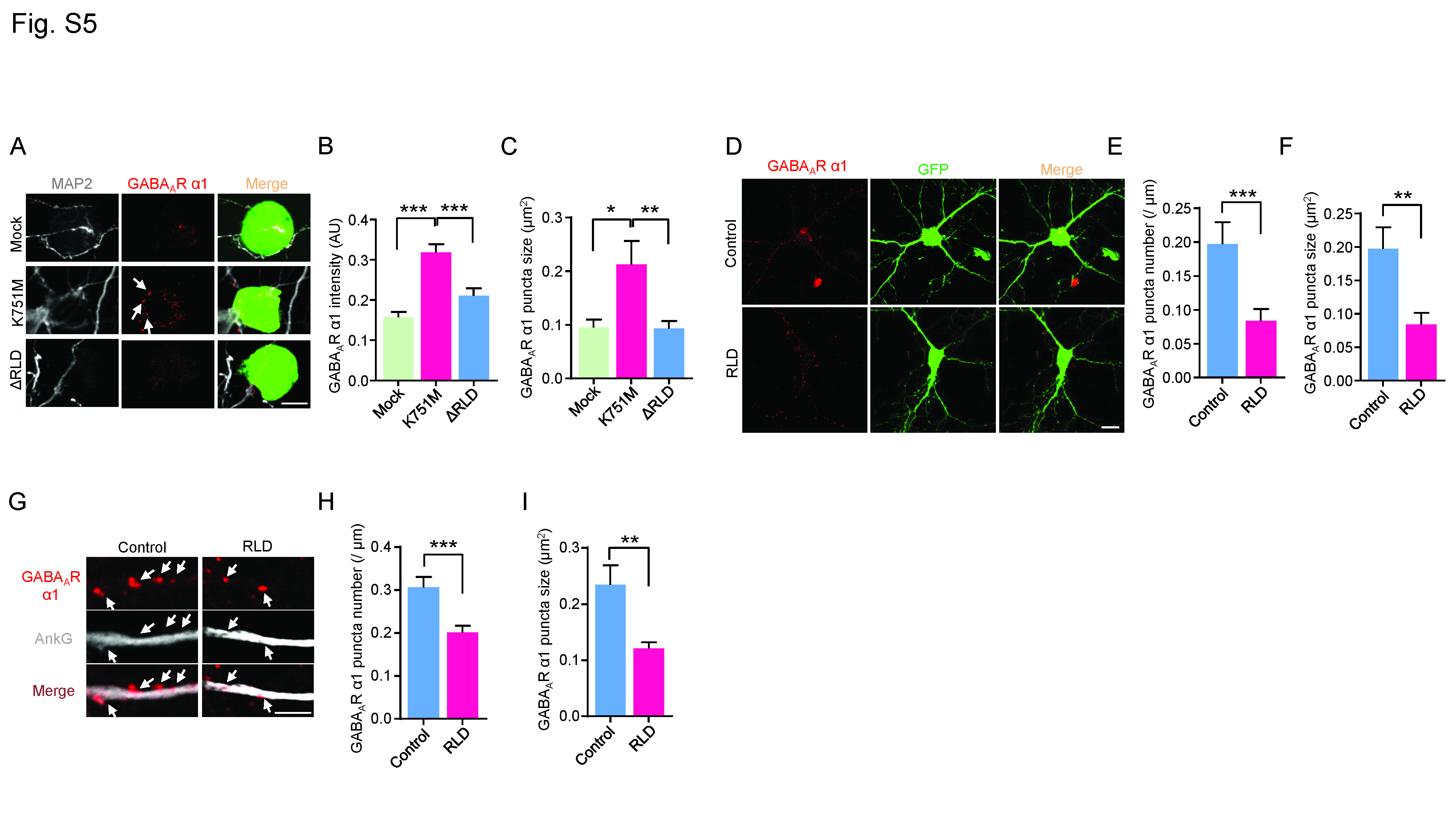

Supplement: Supplementary file 6 — Supplementary Figure 5 [file 41398_2021_1485_MOESM6_ESM.tif]

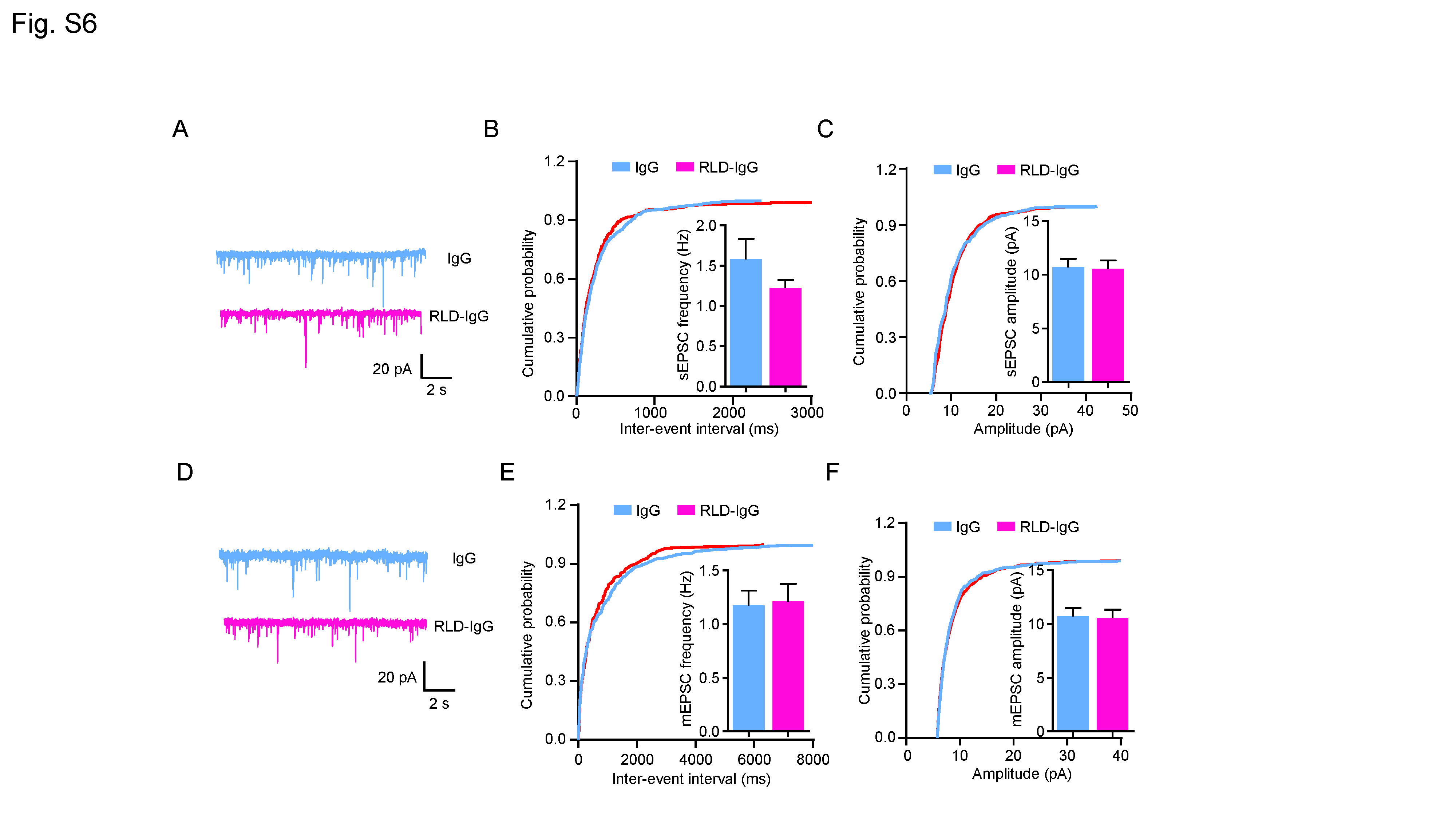

Supplement: Supplementary file 7 — Supplementary Figure 6 [file 41398_2021_1485_MOESM7_ESM.tif]
